# Utilization of Hydrolyzed Agro-Industrial Waste from Arti-Chokes to Obtain Structurally Functional Bacterial Cellulose by *Komagataeibacter rhaeticus* QK23

**DOI:** 10.3390/polym17202783

**Published:** 2025-10-17

**Authors:** Claudio Eduardo Quiñones-Cerna, Gabriela Barraza-Jáuregui, José Alfredo Cruz-Monzón, Fernando Hurtado-Butrón, Bertha Soledad Soriano-Bernilla, Diego Miguel Gutiérrez-Rodríguez, Johnny Huanes-Carranza, Wilmer Ugarte-López, Juan Carlos Rodríguez-Soto, Heber Max Robles-Castillo, Eulalio López-Quiroz, Magaly De La Cruz-Noriega

**Affiliations:** 1 Laboratorio de Biotecnología e Ingeniería Genética, Universidad Nacional de Trujillo, Juan Pablo II Av., Trujillo 13008, Peru; dgutierrez@unitru.edu.pe (D.M.G.-R.); wugartel@unitru.edu.pe (W.U.-L.); hrobles@unitru.edu.pe (H.M.R.-C.); elopezqu3@ucvvirtual.edu.pe (E.L.-Q.); 2 Grupo de Investigación en Biopolímeros, Nanomateriales y Tecnología (GIBINTEC), Facultad de Ciencias Agropecuarias, Universidad Nacional de Trujillo, Juan Pablo II Av., Trujillo 13008, Peru; gbarraza@unitru.edu.pe; 3 Departamento de Ingeniería Química, Universidad Nacional de Trujillo, Juan Pablo II Av., Trujillo 13008, Peru; jcruzm@unitru.edu.pe; 4 Laboratorio Multidisciplinario de Nanociencia y Nanotecnología “Oswaldo Sánchez Rosales, Universidad Nacional de Trujillo, Juan Pablo II Av., Trujillo 13008, Peru; fhurtado@unitru.edu.pe; 5 Laboratorio de Microbiología Ambiental, Universidad Nacional de Trujillo, Juan Pablo II Av., Trujillo 13008, Peru; bsoriano@unitru.edu.pe; 6 Facultad de Ciencias Agrarias, Universidad Privada Antenor Orrego, Av. América Sur 3145, Trujillo 13008, Perú; jhuanesc1@upao.edu.pe; 7 Laboratorio de Citometría, Universidad Nacional de Trujillo, Juan Pablo II Av., Trujillo 13008, Peru; jrodriguezs@unitru.edu.pe; 8 Vicerrectorado de Investigación, Universidad Autónoma del Perú, Lima 15146, Peru; mdelacruzn@autonoma.edu.pe

**Keywords:** biorefinery, biotechnology, lignocellulosic waste, cellulose, fermentation

## Abstract

Bacterial cellulose (BC) is a pure, crystalline biopolymer with broad applications, though large-scale production remains limited by the high cost of culture media. This study evaluated the use of artichoke bract waste as an alternative substrate for BC production by *Komagataeibacter rhaeticus* QK23, focusing on culture optimization and physicochemical characterization of the resulting biopolymer. Infrared spectroscopy revealed functional groups characteristic of cellulose, hemicellulose, lignin, and inulin, along with structural sugars (glucose 24%, xylose 5.07%, arabinose 4.96%, galactose 8.81%, and mannose 1.75%). After hydrolysis with H_2_SO_4_, up to 11.81 g/L of reducing sugars were released and incorporated into Hestrin–Schramm medium lacking glucose. Using a central composite design, inoculum dose (10–20%) and incubation time (7–14 days) were optimized under static conditions at 30 °C. The highest yield (1.57 g/L) was obtained with 20% inoculum after 14 days. The product corresponded to type I cellulose with a crystallinity index of 81.87%, and AFM analysis revealed a surface roughness of 32.96 nm. The results demonstrate that artichoke hydrolysates are a viable and sustainable source for BC production, promoting agricultural waste valorization and cost reduction in industrial biotechnology.

## 1. Introduction

Cellulose is the most abundant natural biopolymer, characterized by its linear D-glucose chains linked by β-1,4 glycosidic bonds, facilitating its crystalline structure and positioning it as a sustainable matrix with broad applicability [1]. Bacterial cellulose (BC) is a water-insoluble exopolysaccharide with the same chemical formula as plant cellulose, produced as a secondary metabolite that forms a solid floating polymeric film on the surface of Kombucha tea during fermentation [2]. BC is noted for its exceptional physicochemical properties, including high crystallinity, tensile strength, water content, porosity, permeability, elasticity, formability, hydrophilicity, low density, degree of polymerization, specific surface area, biocompatibility, purity and biodegradability, offering a promising platform for diverse applications [3].

The production of BC can be modulated by varying the carbon substrates, such as glucose, fructose, and glycerol, as well as stimulants like ethanol in the culture medium [4]. BC production is challenged by the high costs associated with culture media, such as Hestrin–Schramm (HS) medium, in addition to low yield efficiency. These factors render large-scale production unfeasible, as the culture medium represents approximately 50 to 65% of the total production cost [5]. To overcome these limitations, alternative and cost-effective media are being explored, leveraging low-cost and renewable byproducts from agricultural, livestock, textile and other industries [6].

Advances in bioprocessing and media optimization have improved BC production. For example, Heydorn et al. [7] obtained up to 8.2 g/L using industrial by-products such as beet molasses, vinasse, and beer fermentation broth. Additionally, the use of banana peel and celery pomace hydrolysates has been explored, obtaining yields of up to 1.53 g/dm^3^ and 1.79 g/dm^3^ of BC, respectively, using *Gluconacetobacter hansenii* ATCC 23769 [8]. Likewise, Tsouko et al. [9] developed an eco-friendly bioprocess using *Komagataeibacter rhaeticus* and waste products such as wine distillery effluents and biodiesel-derived glycerol, achieving a maximum bacterial cellulose BC production of 9.0 g/L. Therefore, agroindustrial waste demonstrates potential for BC production, with evidence suggesting it also affects the structural and crystalline properties of the biopolymer, opening avenues for adapted BC production from alternative sources.

Vegetable productivity in Peru results in harvests of up to 57,524 tons of artichokes, which anticipates not only greater availability for processing and consumption, but also the presence of a significant amount of waste with an energy source, which currently has not been adequately utilized [10]. During artichoke processing, solid waste is produced, especially the bracts, which are the external of the artichoke flower and constitute approximately 80–85% of the total residual biomass [11]. Kammoun et al. [12] investigated the structural carbohydrate composition of leaf and stem waste of the artichoke species *Cynara scolymus* finding 27% composed of cellulose, 8% hemicellulose and 10% lignin. Likewise, artichoke waste has a high content of fructan-type inulin carbohydrates, which have been found distributed throughout the plant in different proportions. As reported by Zeaiter et al. [13], who investigated the composition of reducing sugars (5%) and inulin (70%).

This study provides an innovative approach to adding value to artichoke bract residues, demonstrating their high availability and limited utilization through biotechnological pathways, as a unique alternative carbon source for BC biosynthesis. Unlike previous studies with conventional residues (molasses, bagasse, or fruit peel) [14], this work integrates a biorefinery approach and process optimization. Therefore, the objective was to determine the effectiveness of using agro-industrial waste from artichoke bracts as an alternative substrate for the production of BC by static fermentation with *Komagataeibacter rhaeticus* QK23, through the optimization of variables such as inoculum dose and incubation time using a central composite design (CCD). Likewise, to characterize the obtained biopolymer in terms of yield, chemical composition, crystallinity, surface morphology, and physical-structural properties using Fourier-transform infrared spectroscopy (FT-IR), X-ray diffraction (XRD) and atomic force microscopy (AFM), to predict its quality and functional potential for biotechnological and sustainable applications.

## 2. Materials and Methods

### 2.1. Obtaining and Characterizing Agroindustrial Waste from Artichoke Bracts (AWAB)

AWAB was collected from agro-industrial production lines in La Libertad, Peru, and disinfected with 100 ppm sodium hypochlorite, rinsed, drained, and dried at 60 °C for 8 h. The dried material was ground, sieved to 0.45 mm, and stored in airtight containers at room temperature [15].

Structural sugars were determined following the National Renewable Energy Laboratory (NREL) protocol described by Woo et al. [16], using an HPLC system equipped (Thermo Scientific, Ultimate 3000, USA) with a charged aerosol detector (Corona Veo-RS) and two specialized sugar columns (SUGAR SP0810 y SUGAR SH-G 6B, Shodex), operating at 60 °C. The mobile phase consisted of a mixture of ultrapure water and acetonitrile (JT Baker, 99.99% purity) at a flow rate of 0.70 mL/min. 300 mg of AWAB was hydrolyzed with 72% sulfuric acid (H_2_SO_4_) at 30 °C for 60 min. Subsequently, the mixture was reduced to 4% H_2_SO_4_ by adding distilled water, and the process was completed in an autoclave at 121 °C for one hour. Samples were then centrifuged at 5000 rpm for 10 min, and the supernatant was filtered through a 0.2 µm polyamide membrane.

The functional groups constituting AWAB were determined by FTIR spectroscopy using a Thermo Nicolet IS50 (USA) in attenuated total reflectance (ATR) mode with a diamond crystal. Measurements were made in the 4000 a 600 cm^−1^ spectrum, with a resolution of 4 cm^−1^ [17].

### 2.2. Hydrolysis of AWAB

A full factorial design considered two independent variables: sulfuric acid concentration (0.5, 2.5 y 5%) and AWAB proportion (0.5, 5.0, 9.0 y 13.0). Hydrolysis was performed at 80 °C for 60 min. Hydrolysates were neutralized with 2 N NaOH to pH 6.8 ± 0.2 before use in fermentation [18].

### 2.3. Evaluation of BC Production Efficiency from AWAB

*Komagataeibacter rhaeticus* QK23 was isolated from Kombucha tea samples and subsequently characterized, as detailed in the previous study by Quiñones-Cerna et al. [19]. The QK23 inoculum was prepared in 50 mL of HS (Hestrin–Schramm) medium containing: Glucose (4.0%), Na_2_HPO_4_ (0.25%), citric acid (0.115%), yeast extract (0.5%) y peptone (0.5%), plus 500 µL/100 mL. To evaluate the impact of incubation time (3–25 days) and inoculum dose (1–20%, v/v) on BC production yield from AWAB hydrolysate, the response surface methodology (RSM). The study used 13 trials based on a quadratic model, as shown in Table 1. Each experiment was conducted in 150 mL flasks containing 20 mL of AWAB hydrolysate supplemented with HS medium without glucose. The initial inoculum, with an optical density of 0.8 to 1.0 at 600 nm, from a 48 h culture at 30 °C.

### 2.4. BC Extraction and Purification

The extracted ABC was washed three times with distilled water and then with 2.5% NaOH (J.T. Baker, Phillipsburg, NJ, USA) at 90 °C for 60 min. A series of rinses with distilled water were performed to adjust the pH to neutral [20]. The BC was then treated with a 2.5% NaClO solution for one hour. To remove any remaining NaClO, successive washes were performed with distilled water. It was then dried for 12 h at 45 °C until constant weight.

### 2.5. Analytical Measurements

Cell biomass was estimated by absorbance (600 nm) using a UV-VIS spectrophotometer (SI-Analytics UviLine 9400, Mainz, Germany). Dry BC weight was measured using an analytical balance (SHS GX600, Tokyo, Japan). At the end of fermentation, the volumetric yield was estimated using the following Equation (1) [21]:

(1) $$\mathrm{Yield}\;\mathrm{BC}\;(g/L)=\frac{\mathrm{BC}\;\mathrm{Dry}\;\mathrm{Weight}\;(g)}{\mathrm{Medium}\;\mathrm{volume}\;(L)}$$

### 2.6. Characterization of BC

#### 2.6.1. FTIR

Dried BC membranes were subjected to FTIR by the attenuated total reflection (ATR) method using a Thermo Nicolet IS50. This facilitated the identification of chemical bonds in the material. Measurements were made in of 4000 a 600 cm^−1^ with a resolution of 4 cm^−1^ [22].

#### 2.6.2. XRD

The crystallinity index (CI) and crystalline area of the BC were calculated using XRD (Rigaku-Miniflex 600) at 30 kV and 20 mA with a wavelength of 0.15 nm. The 2θ scan was performed from 5 to 50 ° at 2 °/min. The CI was determined using Equation (2) [23]:

(2) $$\mathrm{CI}\;=\frac{\;\mathrm{Ac}\;}{\mathrm{Ac}\;+\;\mathrm{Aa}}\;\times \;100$$

where “Ac” is the crystalline area and “Aa” is the amorphous area.

The crystallite size was measured using the Scherrer Equation (3):

(3) $$\mathrm{TCr}\;=\;\frac{\;K\times \lambda }{(\beta \times \mathrm{Cos}\;\theta )}$$

where K = 0.9 is the Scherrer constant, λ the X-ray wavelength, β the full width at half maximum of the peak (radians), and θ the diffraction angle (radians).

#### 2.6.3. AFM

The morphology of the biomaterial was examined by AFM (Bruker Dimension Edge, DE) using tapping mode on 30 × 30 µm scan areas [24]. The Root Mean Square (RMS) surface roughness (Rq), defined as the standard deviation of the z-height values, was quantified with ImageJ 1.52n (USA) and the SurfCharJ plugin following the procedure of Chinga et al. [25].

### 2.7. Statistical Analysis

Data were analyzed using ANOVA and expressed as mean ± standard deviation. RSM analysis was performed using Design Expert 13, with statistical significance set at *p* < 0.05 [26].

## 3. Results and Discussion

### 3.1. Characterization of Agroindustrial Waste

**Figure 1:**
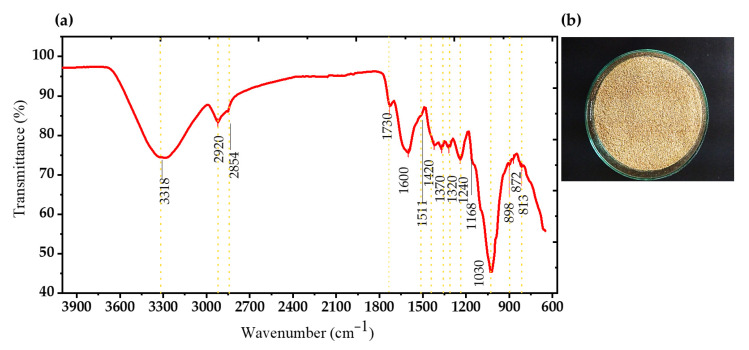
Chemical analysis performed by: (**a**) infrared spectroscopy from (**b**) conditioned AWAB.

In terms of dry matter, the bracts presented an approximate composition of 27% cellulose, 8% hemicellulose, 10% lignin and 55% other compounds (mainly inulin, proteins and ash), according to Kammoun et al. [12] and the results obtained by FTIR and HPLC in this study, confirming their lignocellulosic nature and their potential as a substrate for fermentations. These were qualitatively identified through infrared spectroscopy, as shown in Figure 1. Infrared (IR) spectra around 3318 cm^−1^ were observed, corresponding to the stretching vibration bands of OH groups [27], Moreover, peaks at 2854, 1420, 1030 y 898 cm^−1^ were associated with CH stretching, CH vibration, the C-O-C pyranose ring backbone, and the presence of β-glycosidic bonds, respectively, these groups constitute the cellulose structure [28,29]. Additionally, the band around 1030–1168 cm^−1^ was attributed to the C–O–C stretching of glycosidic bonds [30]. The specific peak at 1030 cm^−1^ belongs to a C-O-C stretching band in the fructofuranose ring, and together with the bands at 872 and 813 cm^−1^, they are classified as β-(2→1) glycosidic bonds from the 2-keto group in fructofuranose [31], these peaks represent the presence of structural inulin, a characteristic component of artichoke [32]. The 2854 cm^−1^ peak corresponds to the C-H stretching vibration in the aldehyde group, which may indicate the presence of cellulose, hemicellulose, and lignin; in turn, the peak near 1511 cm^−1^ may be attributed to lignin vibration in the benzene ring [33]. Other identified peaks include 1420 cm^−1^ associated with the symmetric stretching of the carboxylate group (COO-), found in both fatty acids and amino acids. In addition, the C–N stretching of amide III groups, belonging to proteins, was linked to the band at 13,15 cm^−1^ [34].

**Figure 2:**
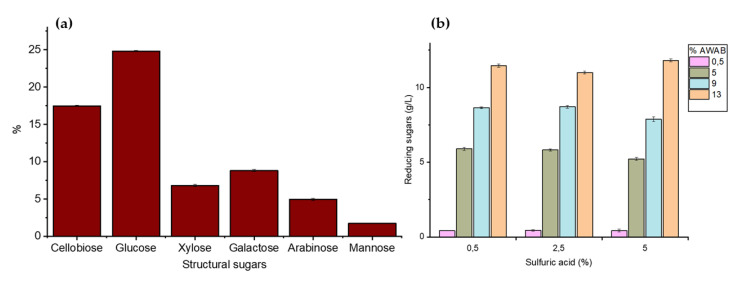
Chemical components determined from AWAB hydrolysis: (**a**) structural sugars and (**b**) evaluation of maximum reducing sugars.

Figure 2a presents the results of the structural sugar characterization of the hydrolyzed pretreated AWAB through HPLC. A high glucose content (24.38 ± 0.004%) was obtained, indicating a high presence of both simple and complex carbohydrates such as cellulose [35], as well as cellobiose (17.48 ± 0.09%), a disaccharide formed by two glucose units, suggesting the existence of cellulose. Xylose (5.07 ± 0.12%), a hemicellulose component, along with arabinose (4.96 ± 0.16%), indicates a significant proportion of hemicelluloses. Galactose (8.81 ± 0.13%) and mannose (1.75 ± 0.02%) point to the presence of other complex polysaccharides, albeit in smaller amounts. These results confirm that artichoke bract residues are rich in cellulose and hemicellulose [36].

Figure 2b, shows the reducing sugars (RS) obtained by hydrolysis at different H_2_SO_4_ concentrations and AWAB proportions (%). A maximum extraction of up to 11.81 ± 0.1 g/L of RS was obtained with 5% H_2_SO_4_ and 13% AWAB; additionally, it was observed that for each H_2_SO_4_ concentration, the RS concentration increases with the AWAB proportion, while the ANOVA analysis shows significant differences in RS production (*p* < 0.0001). However, the H_2_SO_4_ concentration (%, w/v) showed no significant differences between the different concentrations (*p* = 0.996). Therefore, the highest yield of reducing sugars was obtained at 13% artichoke bract residue, regardless of the sulfuric acid concentration (0.5–5%); likewise, pretreatment with concentrated acids improves the hydrolysis of lignocellulosic biomass to obtain fermentable sugars, but it is expensive and dangerous; in contrast, dilute acid hydrolysis is more economical and effective [37]; hence, in this study, a treatment using the lowest concentration of 0.5% H_2_SO_4_ for 13% AWAB was proposed according to the results obtained (Figure 2b).

Furthermore, the maximum yield of 11.81 g/L of reducing sugars representing 18.6% in dry weight (considering 13% of AWAB in 100 mL), a value comparable to the range reported for lignocellulosic residues of sugar bagasse (11.73–43.62%) also using 0.1 N H_2_SO_4_ at 60 min pretreated at different microwave temperatures (50–100 °C) [38]; even exceeds the data obtained by Avila et al. [39] where they reported a 19.1% of reducing sugars obtained at 290 °C, 9 mL/min and 1500 psi from corn slough water, which confirms the efficiency of the dilute acid hydrolysis used.

### 3.2. BC Production from Agroindustrial Waste

Table 2 indicates the maximum BC production yield in treatment 6 with 1.57 ± 0.09 g/L at a 20% inoculum dose and 14 days of incubation, with a cell biomass (OD_600_) of 2.86 ± 0.021 from fermentation of AWAB hydrolysates by *Komagataeibacter rhaeticus* QK23. In turn, in treatments T3 and T4, BC yields were 1.23 g/L and 1.32 g/L, respectively, representing 21% and 16% lower values compared to the maximum yield. It was also observed that a higher inoculum dose (17.2%) slightly increased cell biomass (from 3.07 to 3.10 OD_600_) and also BC yield (from 1.23 to 1.32 g/L); this indicates that, at longer incubation times, a higher inoculum dose can improve both cell growth and BC production. On the other hand, comparing T8 and T10, with the same inoculum dose (10.5%) but different incubation times (25 and 14 days), an increase in cell biomass was observed (from 2.94 to 3.11 OD_600_) with minimal changes in BC yield (from 1.02 to 1.00 g/L). However, the lowest amount was obtained in treatment T7 with 0.3 ± 0.02 g/L BC due to a shorter incubation time of 3 days and an inoculum dose of 10.5%, representing an 80% difference compared to T6.

The results obtained are compared to those reported by Akintunde [40], who achieved 1.6 and 1.4 g/L of BC from enzymatic hydrolysates of sugarcane bagasse under continuous and intermittent agitation, respectively, using Komagataeibacter sp. CCUG73630 at 30 °C for 10 days. Although it exceeds the values obtained by Huang et al. [41], who reached 0.65 g/L BC during agitation (150 rpm) from acid hydrolysate of corn cob using Gluconacetobacter xylinus with 8% inoculum, pH 6.0 and 28 °C for 14 days. Nevertheless, the results differ from those found by Distler et al. [42], who obtained 5.68 g/L of BC using Komagataeibacter intermedius from sulfite liquor. One of these differences is due to the diversity and quantity of sugars extracted from waste, such as glucose, fructose or sucrose, which can stimulate greater biocellulose production [1].

**Figure 3:**
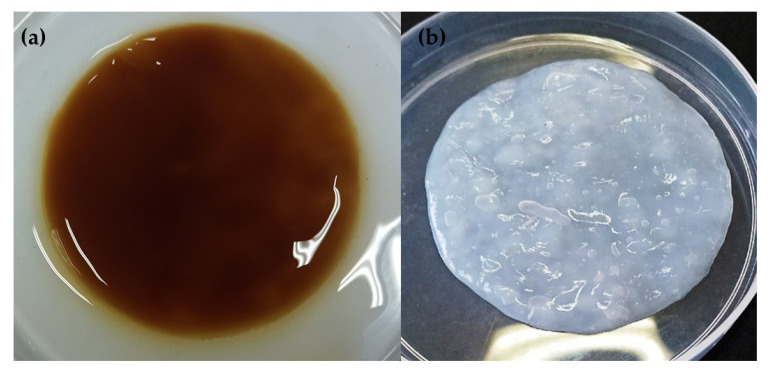
Production of BC by *Komagataeibacter rhaeticus* QK23 from AWAB hydrolysates fermentation. (**a**) Unpurified BC and (**b**) Purified BC.

On the other hand, considering the initial content of 11.81 g/L of reducing sugars, the yield of bacterial cellulose (1.57 g/L) represents a conversion of 13.3%, this result differs from that expressed by Nguyen et al. [43], who obtained 33.3% from hydrolyzed paper waste sludge by *Acetobacter xylinum*, as well as Meng et al. [44], which produces BC at a value of 71.2% in terms of glucose from enzymatic hydrolysates of hemp waste using *Komagataeibacter* spp. In turn, low yields can be affected by the accumulation of formate, acetic acid, furans, and/or phenolic compounds, such as Kim et al. [45], which obtained a 90% reduction in BC due to the presence of 5-hydroxymethylfurfural and furfural from hydrolysates of Miscanthus, barley straw, and pine pretreated with H_2_SO_4_. Therefore, authors suggest the need for additional pretreatments such as detoxification, washing and absorption of inhibitors or concentrating more free sugars for AWAB hydrolysates [46]. Therefore, this study demonstrates that the hydrolysate obtained from AWAB as the sole carbon source favored BC production (Figure 3).

The analysis of variance (ANOVA) showed that the overall model is statistically significant for BC production, with a *p*-value of 0.024, indicating that at least one of the evaluated factors had a significant effect (Table 3). The inoculum dose was the most significant factor (*p* < 0.0001), followed by the incubation time (*p* = 0.0003) and the interaction between both factors was also statistically significant (*p* = 0.002). In contrast, for cell biomass (OD_600_), although the overall model was significant (*p* = 0.026), only the incubation time showed a statistically significant effect (*p* = 0.009), whereas the inoculum dose and the interaction did not exhibit a significant influence on this parameter. These results indicate that BC production is more sensitive to process conditions than cell growth.

**Figure 4:**
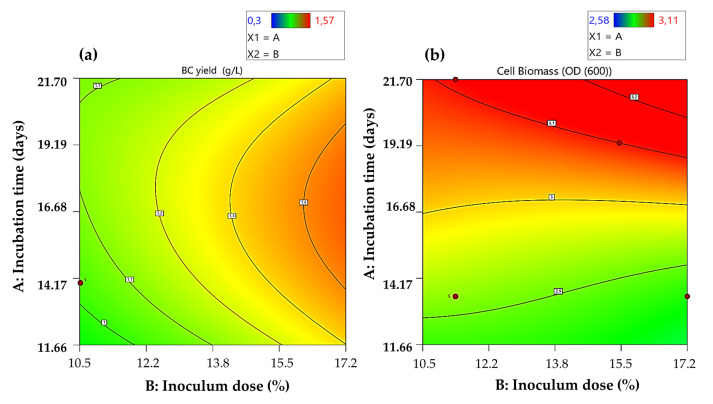
The contour plots show the interaction of the independent variables, highlighting the maximum production of BC (**a**) and biomass (**b**).

Furthermore, in Figure 4a, which corresponds to BC yield, it is evident that increasing the inoculum dose markedly enhances BC production, with a clear upward trend towards the right side of the graph, where warm colors predominate. Incubation time also influences production, although to a lesser extent, as shown by the nearly vertical contour lines. These results correlate with the analysis of variance (ANOVA), which revealed highly significant effects for both inoculum dose (*p* < 0.0001) and incubation time (*p* = 0.0003), with the former being the most determinant factor (F = 310.66). On the other hand, Figure 4b, which represents cell biomass, shows a different pattern where biomass increases with incubation time, while inoculum dose exerts little effect. This behavior aligns with the ANOVA results, where only incubation time was statistically significant (*p* = 0.0094), in contrast to inoculum dose and interaction, which were not. Similarly, the optimization of BC yield and cell biomass showed a fourth-degree polynomial model behavior between the factors and coefficients for a significant model (Equations (4) and (5)).

(4) $$\begin{array}{l} \mathrm{BC}\;\mathrm{yield}\;(g/L)\;=1.02+0.38\;\times \;A+0.24\;\times \;B+0.14\;\times \;AB-0.0015\;\times \;A^{2}-0.19\;\times \;B^{2}-\;\\ 0.13\;\times \;A^{2}B-0.48\;\times \;AB^{2}+A^{3}+B^{3}+0.32\;\times \;A^{2}B^{2}+A^{3}B+AB^{3}+\;A^{4}+B^{4} \end{array}$$

(5) $$\begin{array}{l} Cell\;biomass=2.92-0.025\;\times \;A+0.14*B-0.05\;\times \;AB-0.01\;\times \;A^{2}-0.006\;\times \;B^{2}+\\ 0.05\;\times \;A^{2}B+0.09\;\times \;AB^{2}+A^{3}+B^{3}-0.01\;\times A^{2}B^{2}+A^{3}B+AB^{3}+A^{4}+B^{4} \end{array}$$

When comparing the factors that influence BC production in the present study, Yanti et al. [47] demonstrated that increasing the inoculum dose and incubation time enhances BC production, reporting an optimal value of 25% inoculum and 15 days of fermentation, achieving a maximum yield of 13.85 g/L by *Acetobacter xylinum* LKN6 sing sago liquid waste. Likewise, Aswini et al. [48] demonstrated that a higher inoculum concentration (20%) promoted rapid colonization of the medium and early BC production, in HS medium with glycerol and PEG 6000; furthermore, extending the incubation time up to 30 days allowed the bacteria to maintain cellulose synthesis in the stationary phase, and the combination of both factors resulted in a maximum yield of 469.83 g/L BC wet weight, emphasizing the importance of inoculum control and incubation time in the fermentation process. Similarly, Uğurel & Öğüt [49] showed that the inoculum concentration was the most significant factor (*p* < 0.002), with 8 log UFC/mL being the optimal value that generated a maximum production of 9.1 ± 0.66 g/L (dry weight), significantly higher than those obtained with greater concentrations. Additionally, incubation time also had a significant effect (*p* < 0.001), observing that 14 days was the optimal point before production began to decrease with longer incubations (15–28 days). Therefore, previous studies demonstrate that an optimal inoculum dose combined with an appropriate incubation time maximizes BC yield, highlighting the need for precise control of these parameters to improve fermentation efficiency on an industrial scale.

### 3.3. BC Characterization

**Figure 5:**
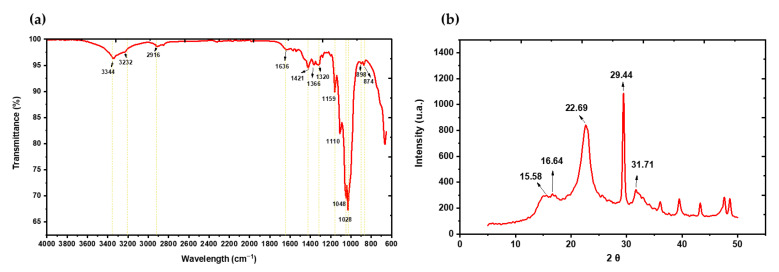
BC from AWAB hydrolysates by *K. rhaeticus* QK23: (**a**) IR spectroscopy, (**b**) XRD.

Figure 5 presents the structural analysis of BC produced by *Komagataeibacter rhaeticus* QK23 from AWAB hydrolysates, using FTIR and XRD. The FTIR spectrum (Figure 5a) revealed bands around 3344 cm^−1^ and 3232 cm^−1^ corresponding to the vibrational stretching of hydroxyl (–OH) groups, indicating a strong presence of intra- and intermolecular hydrogen bonding, characteristic of the cellulose structure [50]. The band at 2916 cm^−1^ is associated with C–H stretching of the methylene groups in the glucose ring [51], while the peak at 1636 cm^−1^ relates to the deformation vibration of water absorbed in the polymer structure [52]. The bands observed at 1421, 1366 and 1320 cm^−1^ are indicative of deformation vibrations of C–H and C–O–H bonds, reflecting the glucose structural composition [53]. In turn, the bands at 1159, 1110 and 1048 cm^−1^, are attributed to the C–O–C stretching vibrations of the glucopyranose ring, and the band at 897 cm^−1^, is characteristic of the β-glycosidic mode (1 → 4) linkage between glucose units, a specific marker of cellulose [54].

Meanwhile, the X-ray diffraction (XRD) analysis (Figure 5b) provides information on the crystallinity of the cellulose obtained in this study. Five main diffraction peaks were observed at 15.58 °, 16.64 °, 22.69 °, 29.44 ° and 31.71 ° (2θ). The peaks at 15.58 ° and 16.64 ° correspond to the crystalline planes (1–10) and (110), while the most intense peak at 22.69 ° corresponds to the (200) crystalline plane, typical of type I cellulose, and in its Iα form [55]. This diffraction pattern is consistent with the production of a high crystallinity cellulose, which is directly related to improved mechanical properties such as tensile strength, elastic modulus and thermal stability [56]. The crystallinity index (CI) of BC in the study was 81.87%, indicating a high degree of structural order within the material, agreeing with other results such as Mouro et al. [57] who obtained around 81.86% CI of BC obtained from the fermentation of raw wastewater from fruit processing using Kombucha microbial consortium at 30 °C for 7 days, although it differs from that obtained by Ziyao et al. [58], who managed to achieve 92.09% CI of BC from restaurant waste, oranges and grapefruits from canteens and supermarkets using *Komagataeibacter sucrofermentans* at 30 °C. The crystallinity level is a relevant parameter as it is directly associated with enhanced properties such as mechanical strength, thermal stability, and a low degree of swelling, desirable characteristics for applications in biotechnology and advanced materials [59]. The crystallite size (TCr) was 3.72 nm, which suggests a finely organized structure at the nanometric level, typical of bacterial cellulose nanofibers [60]. Similarly, the interplanar atomic spacing (d) was measured at 0.68 nm, a value consistent with the interplanar distance of cellulose type I, confirming that molecular orientation favors the formation of crystalline domains [61].

**Figure 6:**
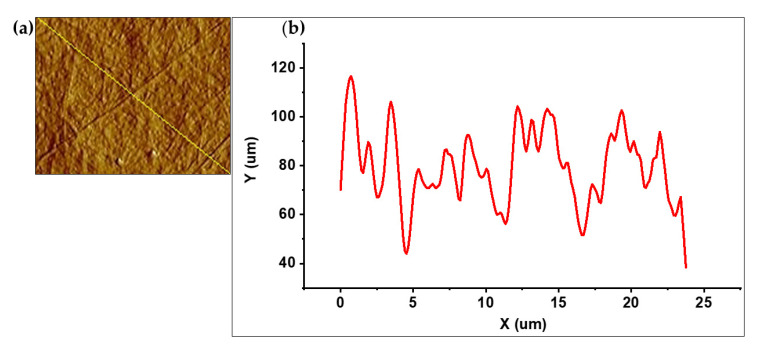
AFM topography of bacterial cellulose obtained from artichoke hydrolysates. (**a**) Three-dimensional image of the microfibrillar surface and (**b**) textural profile corresponding to the diagonal line showing surface height variations.

Figure 6 presents the surface topography characterization of the BC obtained from AWAB hydrolysates using AFM. Figure 6a revealed a surface composed of a randomly oriented interwoven network of microfibrils, with elevated areas and depressions indicating surface morphology heterogeneity. The yellow line traced on the image corresponds to the textural profile represented in the graph (Figure 6b), which shows surface height variations over a distance of approximately 25 µm, where the width of the BC fibers derived from AWH was estimated around 94.77 ± 8.78 nm, being this higher than the range reported by Guhados et al. [62], who reported a fiber diameter range of 27–88 nm of BC from a fructose medium using *A. xylinum* BPR 2001 at pH 5.0 and 28 °C, in a similar manner, Revin et al. [63], reported the average thickness of BC fibrils, measured by AFM, produced in both standard HS and molasses media, ranging from 50 to 90 nm, using *Komagataeibacter sucrofermentans* H-110 under static cultivation at 28 °C for 5 days. The surface roughness, expressed as the RMS value (Rq), was 32.96 nm for the BC derived from AWAB. This parameter, which corresponds to the quadratic mean of height deviations from the average plane, serves as a key indicator of the film’s microstructure [64]. These results compare with those reported by Lopes et al. [65], who described a BC with a smooth surface morphology and a roughness of 21.6 nm from the Hestrin–Schramm medium using *G. xylinum* in static fermentation at 28 °C for 10 days; similarly, it coincides with the results of Quiñones-Cerna et al. [19], who characterized an Rq of 27.05 for BC produced from the fermentation of asparagus waste hydrolysates using *Komagataeibacter rhaeticus*. Therefore, these results suggest that the BC produced from artichoke hydrolysates possesses a suitable surface morphology for functional applications, without compromising its structural integrity, as corroborated by the preceding crystallinity and molecular structure analyses.

Therefore, the structural features observed in this study highlight the functional potential of BC for various advanced applications. The high crystallinity index (81.87%) indicates an ordered molecular organization, which enhances tensile strength, thermal stability, and low swelling capacity, making it advantageous for the formulation of biomedical scaffolds and food packaging films that require stability under humid conditions [66]. Likewise, the estimated nanofibrillar network, with a width of around 95 nm and an Rq of 32.96, contributes to a good specific area and a recommended microtopography that can promote cell adhesion and proliferation in dressings and scaffolds used in tissue engineering [67]. Similarly, heterogeneous surface morphology can increase particle absorption and entrapment, providing utility in the environmental area with water purification systems and filtration membranes, such as Mir et al. [68], who developed BC and graphene oxide composite as water filtration membranes. Overall, the combination of high crystallinity, nanometric fibrillar structure, and moderate roughness in the BC obtained from artichoke hydrolysates highlights its versatility for applications in biomedicine, packaging, and filtration technologies.

## 4. Conclusions

Hydrolysates derived from artichoke bract waste constitute an effective and sustainable carbon source for bacterial cellulose production by *K. rhaeticus* QK23. The implementation of the central composite design allowed for the optimization of culture conditions, identifying an inoculum dose of 20% and an incubation period of 14 days as key factors for maximizing yield, achieving a production of 1.57 g/L of dry weight bacterial cellulose. The characterization of the obtained biopolymer confirmed its functional properties, evidenced by the presence of bands typical of type I cellulose (FTIR), a high crystallinity index of 81.87% (XRD) and a homogeneous fibrillar network with a surface roughness of 32.96 nm (AFM). These findings not only validate the technical and structural feasibility of the produced biopolymer but also highlight the valorization potential of a low-cost agricultural residue through an eco-efficient biotechnological process, contributing to the development of functional materials derived from renewable sources.

## Figures and Tables

**Table 1:** Values applied according to the rotating central composite design for two independent variables.

Coded Levels					
	−1.4	−1	0	1	+1.4
Incubation Time (days)	3	6	14	17	25
Inoculum Dose (%)	1.0	3.8	10.5	17.2	20.0

**Table 2:** BC yield and cell biomass from fermentation of AWAB hydrolysates.

Treatment (T)	Inoculum Dose (%)	Incubation Time (Days)	Cell Biomass (OD_600_)	BC Yield (g/L)
1	3.8	6	2.58 ± 0.011	1.27 ± 0.05
2	17.2	6	2.81 ± 0.012	0.80 ± 0.02
3	3.8	22	3.07 ± 0.030	1.23 ± 0.21
4	17.2	22	3.10 ± 0.013	1.32 ± 0.02
5	1	14	2.93 ± 0.026	0.48 ± 0.05
6	20	14	2.86 ± 0.021	1.57 ± 0.09
7	10.5	3	2.70 ± 0.017	0.30 ± 0.02
8	10.5	25	3.11 ± 0.044	1.00 ± 0.08
9	10.5	14	2.90 ± 0.018	0.97 ± 0.19
10	10.5	14	2.94 ± 0.007	1.02 ± 0.05
11	10.5	14	2.98 ± 0.015	1.05 ± 0.13
12	10.5	14	2.82 ± 0.008	1.02 ± 0.07
13	10.5	14	2.95 ± 0.012	1.08 ± 0.27

**Table 3:** ANOVA of BC and Biomass in AWAB hydrolysate fermentation by *K. rhaeticus* QK24.

Response	BC (g/L)			Cell Biomass (OD600)		
Source	Sum of squares	F	*p*	Sum of squares	F	*p*
Model	1.35	5.33	0.0245	0.2674	8.75	0.02
A-Inoculum dose	0.5868	310.66	<0.0001	0.0025	0.6414	0.47
B-Incubation Time	0.2473	130.94	0.0003	0.084	22	0.009
AB	0.0784	46.95	0.0024	0.01	2.62	0.18
Pure Error	0.0067			0.01		
Cor Total	1.36			0.28		

## Data Availability

Data is available within the article.
